# *Notes from the Field*: Emergence of an Mpox Cluster Primarily Affecting Persons Previously Vaccinated Against Mpox — Chicago, Illinois, March 18–June 12, 2023

**DOI:** 10.15585/mmwr.mm7225a6

**Published:** 2023-06-23

**Authors:** Emily A. G. Faherty, Taylor Holly, Yasmin P. Ogale, Gordon Crisler, Ashley Becht, David Kern, Lavinia Nicolae, Hillary Spencer, Michael Wasz, Janna L. Kerins, Alyse Kittner, Amber Staton, Carla Hardnett, Christina Hutson, Crystal M. Gigante, Laura Quilter, Ian Kracalik, Stephanie Black, Andrea M. McCollum, Agam K. Rao, Irina Tabidze

**Affiliations:** ^1^Epidemic Intelligence Service, CDC; ^2^Chicago Department of Public Health, Chicago, Illinois; ^3^Division of Sexually Transmitted Diseases Prevention, National Center for HIV, Viral Hepatitis, STD, and TB Prevention, CDC; ^4^Division of High-Consequence Pathogens and Pathology, National Center for Emerging and Zoonotic Infectious Diseases, CDC.

During April 17–May 5, 2023, 13 monkeypox (mpox) cases were reported to the Chicago Department of Public Health (CDPH) after 2 months during which only a single case had been reported. The cluster was remarkable because it comprised more than 10 cases at a time when sporadic cases or small clusters (i.e., involving fewer than three cases) were being reported in the United States, and >69% of the persons in this cluster had received 2 doses of JYNNEOS or 1 dose of ACAM2000 vaccine.* Some cases among persons who received doses of JYNNEOS vaccine are expected to occur based on vaccine effectiveness data (*1*,*2*); however, the observed proportion of cases among persons who had received 2 doses of JYNNEOS or 1 dose of ACAM2000 in this cluster was unusual. This increase in cases before large summer events scheduled nationwide and in Chicago raised concerns about possible future case increases. 

On May 9, 2023, CDPH issued a health alert,^†^ urging clinicians to remain vigilant for mpox cases and encouraging vaccination for persons at risk for mpox.^§^ CDPH and CDC launched an investigation to 1) determine the cluster’s scope and etiology by evaluating patients’ commonalities, JYNNEOS^¶^ vaccine cold-chain management, whole genome sequencing of clinical samples, and serologic immune response after infections, and to 2) identify important risk factors for mpox exposure to guide prevention efforts. This activity was reviewed by CDC and was conducted consistent with applicable federal law and CDC policy.**

During March 18–June 12, 2023, 40 laboratory-confirmed mpox cases were identified in Chicago, including 22 (55%), five (13%), and 13 (33%), respectively, among patients who had received 2 doses of JYNNEOS or 1 dose of ACAM2000 vaccine, those who had received 1 vaccine dose of JYNNEOS vaccine, and those who had not received any vaccines for mpox (Table). All cases occurred among persons who were assigned male sex at birth; 37 (93%) identified as male and 28 (70%) as gay. Median age was 33 years (IQR = 23–49 years), and non-Hispanic White men accounted for the largest percentage of patients (19; 48%), followed by Hispanic or Latino (eight; 20%) and non-Hispanic Black or African American men (seven; 18%). Eleven (28%) patients were living with HIV, 10 of whom had received 2 doses of JYNNEOS or 1 dose of ACAM2000 vaccine and whose HIV was well-controlled (CD4 count >200 cells/mm^3^ and viral load <200 viral copies/mL). Three (8%) patients experienced concurrent sexually transmitted infections at the time of mpox diagnosis.

**TABLE:** Characteristics of patients with mpox, by vaccination status (N = 32) — Chicago, Illinois, March 18–June 12, 2023

Characteristic	No. (%)	
	Persons who received 2 doses of JYNNEOS vaccine or 1 dose of ACAM2000* (n = 22)	Persons who received 1 dose of JYNNEOS vaccine/Unvaccinated (n = 18)
**Median age, yrs (IQR)**	34 (31–40)	30.5 (28–38)
**Current gender identity**		
Male	20 (91)	17 (94)
Unknown	2 (9)	1 (6)
**Sexual orientation**		
Gay	17 (77)	11 (61)
Bisexual	1 (4.5)	3 (17)
Other	1 (4.5)	0 (—)
Unknown	3 (14)	4 (22)
**Race and ethnicity^†^**		
Asian, non-Hispanic	1 (4.5)	0 (—)
Black or African American, non-Hispanic	1 (4.5)	6 (33)
White, non-Hispanic	13 (59)	6 (33)
Hispanic or Latino	4 (18)	4 (22)
Other, non-Hispanic	3 (14)	1 (6)
Unknown	0 (—)	1 (6)
**Persons living with HIV**	5 (23)	6 (33)
**Persons hospitalized for mpox**	0 (—)	2 (11)
**Persons who received tecovirimat for mpox**	6 (27)	2 (11)
**Persons who reported concurrent sexually transmitted infections^§^**	1 (5)	2 (11)
**Persons who reported attending an event^¶^ 3 weeks before symptom onset**	6 (27)	3 (17)
Median no. of sex partners** (range)	3 (1–20)	1 (0–6)

**Abbreviation**: mpox = monkeypox.

* Persons in this group had received 2 doses of the JYNNEOS vaccine or 1 dose of ACAM2000 >2 weeks before illness onset by either subcutaneous or intradermal administration route.

^†^ No persons identifying as Native Hawaiian or other Pacific Islander or American Indian or Alaska Native were reported.

^§^ Including syphilis and gonorrhea.

^¶^ Patients were asked if they attended any large public or private events in the 3 weeks preceding symptom onset (i.e., concerts, weddings, parades, or festivals).

** Number of sexual partners reported in the 3 weeks preceding symptom onset.

Most vaccinated patients in this cluster received 1 or 2 JYNNEOS vaccine doses during July–August 2022; the timing of vaccination is similar to an mpox cluster in France involving vaccinated persons who acquired mpox >6 months after vaccination^††^ (*3*). In the Chicago cluster, the median interval from receipt of the second JYNNEOS vaccine dose to mpox diagnosis was 8.4 months (IQR = 7.9–8.8 months). Among the 22 patients who received 2 doses of JYNNEOS vaccine or 1 dose of ACAM2000, eight (36%) received 2 subcutaneous doses of JYNNEOS, seven (32%) received 1 subcutaneous and 1 intradermal dose, one (5%) received 2 intradermal doses, and one received 1 dose of ACAM2000^§§^ abroad.^¶¶^ In discussions with Bavarian Nordic (the vaccine manufacturer) and the Strategic National Stockpile (SNS),*** CDC and CDPH identified no concerning temperature excursions that could result in reduced vaccine effectiveness among involved vaccine lots at SNS, during transit from SNS to CDPH, or in CDPH custody. Patients were vaccinated at multiple locations, and CDPH is investigating potential temperature excursions in transportation, storage, and handling at vaccination sites.

Preliminary medical record review indicates that vaccinated patients experienced self-limited illness, managed in outpatient settings. Compared with patients who received 2 doses of JYNNEOS or 1 dose of ACAM2000 vaccine, patients who received 1 dose of JYNNEOS or no vaccines experienced a higher prevalence of lesions affecting the genital (43% versus 6%) and ocular (29% versus none) mucosa. The two hospitalized patients in this cluster had not received any mpox vaccine and had advanced HIV (<200 CD4 cells/mm^3^). Preliminary sequencing results from one unvaccinated patient and three patients who had received 2 doses of JYNNEOS or 1 dose of ACAM2000 vaccine indicate that *Monkeypox virus* (MPXV) among these Chicago patients is consistent with the B.1 variant of clade IIb MPXV, the predominant variant during the 2022–2023 outbreak. Genomic sequences revealed very few point mutations relative to published MPXV genomes, with no changes predicted to cause amino acid changes or increased pathogenicity. In terms of risk factors, patients who received 2 doses of JYNNEOS or 1 dose of ACAM2000 had a median of three sex partners (range = one to 20) during the 3 weeks before symptom onset, compared with 1.5 (range = 0–6) among patients who received 1 dose of JYNNEOS vaccine and unvaccinated patients. 

Although the cause of this cluster has not yet been determined, leading hypotheses include a potential high number of sexual exposures in a network with many vaccinated persons, decreased vaccine effectiveness due to waning of humoral immunity, or vaccine mishandling or administration errors. Health departments are encouraged to report vaccination status of mpox patients to CDC for rapid detection of similar clusters among persons who were previously vaccinated. Persons with known or suspected mpox exposures should isolate and seek testing if they develop mpox symptoms, even if they have been vaccinated. Temporary sexual behavior changes, such as limiting the number of new or multiple sex partners and limiting sex in settings where anonymous sexual contact with multiple partners occurs can also help prevent mpox. Persons eligible for vaccination, particularly those with advanced HIV and other immunocompromising conditions, should receive 2 doses of JYNNEOS vaccine. Additional research on the durability of JYNNEOS vaccine–induced immunity is needed.

## Footnotes

* Persons in this group had received 2 doses of JYNNEOS or 1 dose of ACAM2000 vaccine >2 weeks before illness onset by either subcutaneous or intradermal administration route. One patient received 1 dose of ACAM2000 while in the United Kingdom.

†
https://www.chicagohan.org/alert-detail/-/alert-details/46678186?p_r_p_categoryId=undefined

§ Persons recommended to receive mpox vaccination include gay, bisexual, or other men who have sex with men and transgender, nonbinary, or gender-diverse persons who during the previous 6 months have had a new diagnosis of one or more sexually transmitted disease (e.g., chlamydia, gonorrhea, or syphilis) or who have had more than one sex partner. In addition, mpox vaccination is recommended for anyone who has experienced or anticipates experiencing any of the following scenarios: had a known or suspected exposure to someone with mpox, had a sex partner during the previous 2 weeks who was diagnosed with mpox, had sex at a commercial sex venue (e.g., a sex club or bathhouse) during the previous 6 months, had sex during the previous 6 months at a large commercial event or in a geographic area (e.g., city or county) where *Monkeypox virus* transmission is ongoing, or had sex in exchange for money or other items during the previous 6 months.

¶
https://www.fda.gov/media/131078/download

** 45 C.F.R. part 46.102(l)(2), 21 C.F.R. part 56; 42 U.S.C. Sect. 241(d); 5 U.S.C. Sect. 552a; 44 U.S.C. Sect. 3501 et seq.

†† Persons received 1–2 doses of JYNNEOS (also known by the brand name Imvanex).

§§
https://www.fda.gov/files/vaccines%2C%20blood%20%26%20biologics/published/Package-Insert---ACAM2000.pdf

¶¶ Counts of doses by mode of administration might not sum to the total due to missing data from vaccines received out of jurisdiction.

*** Collaboratively managed by the U.S. Department of Homeland Security and CDC.

## References

[1] Dalton AF, Diallo AO, Chard AN, ; CDC Multijurisdictional Mpox Case Control Study Group. Estimating effectiveness of JYNNEOS vaccine in preventing mpox: a multijurisdictional case-control study—United States, August 19, 2022–March 31, 2023. MMWR Morb Mortal Wkly Rep 2023;72:553–8. 10.15585/mmwr.mm7220a3 37200229 PMC10205167

[2] Deputy NP, Deckert J, Chard AN, Vaccine effectiveness of JYNNEOS against mpox disease in the United States. N Engl J Med 2023;NEJMoa2215201. 10.1056/NEJMoa2215201 37199451 PMC10962869

[3] Jamard S, Handala L, Faussat C, Resurgence of symptomatic mpox among vaccinated patients: first clues from a new-onset local cluster. Infect Dis Now 2023;53:104714. 10.1016/j.idnow.2023.104714 37120092 PMC10156087

